# Supplementary material for: Unraveling the hidden universe of small proteins in bacterial genomes
Source: Mol Syst Biol. 2019 Feb 22;15(2):e8290. doi: 10.15252/msb.20188290 (PMC6385055; doi:10.15252/msb.20188290)

**APPENDIX**

**TABLE of CONTENTS:**


**0. <u>INDEX OF DATASETS</u>** (page 2)

**1. <u>SUPPLEMENTARY INFORMATION</u>**

1.1- Detection of new proteins in a minimal genome: signal comparative between experimental methodologies, conservation and RanSEPs in Mycoplasma pneumoniae (page 3)

1.2- RanSEPs in detecting standard size proteins and DISCO-Bac comparative (page 5)

1.3- Ribosome profiling in Escherichia coli (page 6)

1.4- Computational performance of RanSEPs in comparison with other desktop tools (page 7)

1.5- RanSEPs in a multi-species context (page 8)

1.6- SEP probability per GC content (page 9)

1.7- Phobius prediction assessment (page 10)

**2. <u>SUPPLEMENTARY FIGURES LEGENDS</u>** (page 12)

**3. <u>SUPPLEMENTARY FIGURES</u>** (page 17)

## 0. INDEX OF DATASETS

1. **Dataset EV1**. Results for protein searches in 116 shotgun MS experiments in M. pneumoniae.
2. **Dataset EV2**. Mass spectrometry and RNA-Seq condition, name and access references.
3. **Dataset EV3.** *M. pneumoniae* database, associated RanSEPs scores and its "–omics" related information.
4. **Dataset EV4.** Heavy peptides tested in *M. pneumoniae.*
5. **Dataset EV5.** Detection comparison including 6 Mycoplasma species.
6. **Dataset EV6.** Results for *M. genitalium.*
7. **Dataset EV7.** Results for *M. hyopneumoniae.*
8. **Dataset EV8.** Results for *M. capricolum.*
9. **Dataset EV9.** Results for *M. gallisepticum.*
10. **Dataset EV10.** Results for *M. mycoides.*
11. **Dataset EV11.** Results for *E.coli.*
12. **Dataset EV12.** Results for *P. aeruginosa.*
13. **Dataset EV13.** Results for *S. aureus.*
14. **Dataset EV14.** Results for *L. lactis.*
15. **Dataset EV15.** Results for *Synechocystis.*
16. **Dataset EV16.** Results for *H. pylori.*
17. **Dataset EV17.** SEPs detected in this work.
18. **Dataset EV18.** SEPs used for the validation and method comparative.
19. **Dataset EV19.** Prediction quality metrics for 6 annotation prediction tools.
20. **Dataset EV20.** Total numbers after running RanSEPs in 109 bacterial genomes.
21. **Dataset EV21.** ncRNAs that could encode for SEPs.
22. **Dataset EV22.** Functional study of SEPs.
23. **Dataset EV23.** Description of the output generated by RanSEPs.

# 1. SUPPLEMENTARY INFORMATION

## 1.1 Detection of new proteins in a minimal genome: signal comparative between experimental methodologies, conservation and RanSEPs in *Mycoplasma pneumoniae*

We validated the predictions of RanSEPs for *M. pneumoniae* by integrating results from RNAseq, shotgun MS experiments, and conservation data (Figure S12A). Considering that the smallest protein detected by shotgun MS (with 1 UTP) was 20 amino acids in length, we reduced the database size to 11,858 putative ORFs ≥ 20 amino acids (Datasets EV1 and EV2). Using this database, RanSEPs predicted 756 ORFs: 612 standard proteins (598 annotated and 14 new) and 144 SEPs (26 annotated and 118 new).

Of the 144 predicted SEPs, we confirmed 28 by shotgun MS with ≥ 2 UTPs (7 new and 21 annotated proteins) (Figure S12A). Of these, 16 were conserved in other bacteria and had RNA levels above the threshold (log2(counts) ≥ 4.5). On the other hand, the remaining 12 were not conserved, and one of these did not pass the RNA expression threshold. Interestingly, the number of potential new SEPs is increased by three when considering the positively scored smORFs that were detected by MS with 1 UTP.

Twenty-seven of the 116 predicted SEPs not detected by MS were transcriptionally active (23 new and 4 annotated). Regarding these transcriptionally active SEPs, 19 of the 23 new ones did not overlap with any other transcriptionally active annotated element.

Another group included those SEPs predicted by RanSEPs but not conserved or associated with RNA or MS (83 new and 1 annotated proteins; of which 77 did not

overlap in more than 25% of their length with annotated genes), and 51 presented a low-expression profile (≥2 log2(counts)). Out of the 77 SEPs, 26 had at least half of their sequence repeated in other regions of the genome. In such cases, besides having a lower number of UTPs, any repeated RNAseq reads would be discarded during analysis and thereby result in an underestimation of RNA levels (i.e., levels below the detection threshold). Some of these partly duplicated smORFs could be pseudogenes arising from recombination or stop codons. Furthermore, it is important to note that at least three of the SEPs correspond to a larger protein in *M. genitalium* and are the result of a stop codon introduced in the homologous gene in *M. pneumoniae.*

For those putative smORFs that were not predicted by RanSEPs but had significant RNA expression levels (3,683 smORFs) or were conserved (145 smORFs), we found that 90% and 100% of them overlapped with other genes, respectively (Figure S4A and S4B).

RanSEPs did not predict 65 of the annotated standard proteins of *M. pneumoniae*. Of these, we detected 13 (20%) by MS, for which the RanSEPs scores were between 0.7 and our threshold of 0.85 (Figure S10A). As such, with a more permissive threshold, we would have been able to detect these proteins, but as a consequence, there would have been a greater number of false positives (FPs). On the other hand, RanSEPs predicted 14 novel standard proteins, all with sizes between 100 and 200 amino acids, of which we were only able to detect four by MS (Figure S13B).

A lack of high responsive UTPs (HR_UTPs) could explain the cases where SEPs and standard proteins were predicted by RanSEPs but not found by MS. To test this hypothesis, we ran PeptideSieve on the database (Dataset EV3). We found that both the

annotated and new proteins that were predicted by RanSEPs and detected by MS, had a significantly higher number of HR_UTPs compared to those RanSEPs positives that were not detected by MS (p-value=1e-6, Figure S12B). Only one out of the 116 predicted novel SEPs not detected by MS had ≥ 2 HR_UTPs. Of the 10 standard proteins that were scored as positive by RanSEPs but not found by MS, none of them presented ≥ 2 HR_UTPs. The fact that these proteins had a greater hydrophobic profile compared to the proteins detected by MS (p-value=0.0003) could explain why they were not present in any MS experiment. In general, novel proteins presented a low number of detectable UTPs (Figure S12C). Thus, although some predicted proteins could be transcriptionally active, conserved and with ≥ 2 UTPs, their peptides could have properties that hamper detection by MS. This result shows why it is difficult to corroborate the existence of some proteins by MS experiments.

**1.2. RanSEPs in detecting standard size proteins and DISCO-Bac comparative**

Using *M. pneumoniae* as a reference, we tested the precision of RanSEPs as tool for general annotation (no discrimination by size) and compared it to other tools. RanSEPs was ranked as the best tool as it properly predicted 26 out of 27 known SEPs in *M. pneumoniae*. BASys, GeneMarkS and Prodigal provided positive predictions for only 18 SEPs, while CPC and Glimmer were unable to detect any (Figure S14A). Likewise, our software correctly predicted 598 out of 662 annotated standard proteins (90.3% accuracy), while the other tools achieved accuracies ranging between 23.9% (BASys) and 76.6% (Prodigal). This test was performed ensuring that the target annotated protein was not considered in the training process.

RanSEPs was the only tool that correctly predicted the 4 new standard proteins discovered in this work and the 8 new SEPs (Figure S14B). The two SEPs that appeared in non-targeted MS experiments with 1 UTP and not in targeted MS experiments were not predicted as positive by any of the methods. The high level of success of RanSEPs was not due to an excess of positively scored annotations (Dataset EV19). Furthermore, we generated a set of true negatives (TNs) from putative ORFs and smORFs of *M. pneumoniae* that were not found in its close relative *M. genitalium*, as well as a true positive set (TPs) based on MS data.

We further validated RanSEPs by comparing it with the DISCO-Bac study performed in 17 putative smORFs of *Helicobacter py*lori (Friedman et al. 2017). Five SEPs were identified by targeted MS, 2 by DISCO-Bac and 4 predicted by RanSEPs. The detected peptide for the SEP with a negative score in both tools was shared with a standard protein. Regarding the rest, whereas three were misclassified as coding by DISCO-Bac, none were retrieved by our tool (Dataset EV18, Figure S15).

**1.3. Ribosome profiling in *Escherichia coli***

In this analysis we explored the relationship between proteins found by MS and their ribosome profiling coverage in *E. coli* in order to support SEPs detected by MS. We used two different ribosome profiling datasets including 5 samples in total. First dataset was the one presented by Hücker et al. 2017 (accession: SRP113660), including 6 samples covering 3 different conditions (3 RNASeq and 3 RiboSeq samples). Second dataset was presented in Jing W. et al. 2014 (accession: E-MTAB-2903), specifically two replicates in wild type conditions. To process each dataset, we aligned the reads to the E. coli K12 genome using Bowtie2 and allowing 1 mismatch (Langmead and

Salzberg 2012). Later, we filtered the alignment with SAMtools (Li et al. 2009) to select paired reads mapped unambiguously with a minimum alignment quality of 30. The final step required BEDtools to extract the coverage. We used the ratio between RPKM (Read per Kilobase Million) coverage derived from RiboSeq and the same value derived from RNASeq (abbreviated as RCV) and then standardized the distribution by feature scaling (min-max standardization). It is important to remark, that every annotation with RPKM below 0.2 (both for RPKM associated to ribosomes and in general) and/or a RCV<0.2 were considered to have RCV=0.0 (Dataset EV11). This value was computed for the 5 different samples that we merged taking the mean value for each gene. Then, only genes that did not overlap with any other known annotation were selected to avoid the expression signal coming from different overlapping frames. Statistical analysis was supported by a non-parametric test (Mann-Whitney rank test) as normality of distributions was not satisfied (tested by Shapiro-Wilk). We used this approach to evaluate differences in the distributions of RCV grouped by number of UTPs (Figure S5) or by RanSEPs scores (Figure 3C).

### 1.4. Computational performance of RanSEPs in comparison with other desktop tools

For this comparison, we ran the same tests for RanSEPs, glimmer and prodigal. CPC, BASys and GeneMarkS were not considered in the comparison as they are web services for which queue systems are required to run their applications and this provides an inaccurate representation of their performances. The test performed consisted in running each tool with 8 bacterial genomes ranging in genome size from 0.5Mb to 9.1Mb:

*Mycoplasma genitalium* (0.58Mb), *Mycoplasma mycoides* (1.15Mb), *Helicobacter pylori* (1.56Mb), *Lactobacillus lactis* (2.39Mb), *Bacillus subtilis* (4.09Mb), *Escherichia coli* (5.23Mb), *Pirellula staley* (6.53Mb), and *Bradyrhizobium japonicum* (9.1Mb). RanSEPs was used with three different configurations: 1, 5 and 25 folds. With this, we showed that although RanSEPs requires more time because of the Blast computation and its iterative behavior, its performance is still comparable to the rest of tools and this increased time can be justified by the significant increase in prediction accuracy. We compared the different tools in terms of computational time required to provide the prediction (Figure S14A), and in terms of CPU usage (Figure S14B). In terms of CPU, RanSEPs performed similar to the other tools with the exception of a high CPU requirement during the execution of Blast to define the training sets.

In order to provide faster predictions, RanSEPs allows to pass an already generated database in previous runs that improves the performance significantly (execution time <5 minutes for the 109 bacterial species considered). Argument '-db' included in the tool allows to activate this behavior.

### 1.5. Running RanSEPs in a multi-species context

When running RanSEPs in 109 genomes using the default configuration extracted from *M. pneumoniae* we observed that for organisms with higher number of SEPs more accurate predictions were extracted (higher TPR, lower FPR) maximizing the number of true SEPs in the positive set (85% of proteins in the set being SEPs) and increasing the negative set size (this configuration is not applicable to organisms with low number of annotated SEPs as set size would be too small). We recommend to follow the procedure

to set parameters described in the original manuscript in order to optimize the predictions in a specific genome of interest. Despite this fact, default configuration provided good average results in terms of TPR and results can be use to guide new MS searches or validate experimental results.

On the other hand, after running RanSEPs in 109 bacterial species we observed that the state of the original annotation has impact in the predictions. As example, we observed that prediction of SEPs was significantly higher in *Escherichia coli* strains K12 and O157:H7. We studied this case and we observed that original annotation was biasing our expectations of 'new' SEPs that were in reality known SEPs described in *E. coli* CFT073. K12 and O157:H7 strains had recorded 225 and 326 SEPs, respectively, and all the SEPs with clear homology with O157:H7 were predicted as positives in this strain. Those SEPs, in addition to the new predicted ones, make this strains to present a higher increment in coding SEPs. This problem is not easy to approach but RanSEPs allows to provide a custom organism to use as reference for homology instead of using the internal 109 NCBI genomes database. In addition, results can be prioritized by the presence of Ribosome Binding Sites and homology.

### 1.6. SEP probability per GC content

In order to assess whether the number of SEPs in an organism is maximized for GC contents closer to 40%, we used the set of probabilities shown below.

Assuming that the probability of each nucleotide corresponds to:

$$P\,(A) = P\,(T) = \frac{100 - \%GC}{2}$$

the probability of a specific codon can be computed as:

$$P\,(CODON = IJK) = P\,(I)\,xP\,(J)\,xP\,(K)$$

Consequently, the probability to find a start or a stop (considering translation tables 4 and 11):

$$P\ (START) = P\ (ATG) + P\ (GTG) + P\ (TTG) P\ (STOP_4) = P\ (TAG) + P\ (TAA)$$

$$P\ (STOP_{11}) = P\ (STOP_4) + P\ (TGA)$$

Thus, the probability of any other codon other than a stop codon is:

$$P\ (CODON \neq STOP) = 1 - P\ (STOP)$$

Finally, the probability to find a SEP (9 to 99 amino acids+starting methionine) for a specific GC content is:

$$P\ (SEP) = P\ (START) x P\ (STOP) x \sum_{L=9}^{99} P\ (CODON \neq STOP)^L$$

This value is corrected for by subtracting the P(SEP<=9) as we assume that no protein is going to be shorter than that.

Knowing this, each %GC content will present a bias in the number of start and stop codons (Figure S16A) and this will directly affect the probability to find a SEP (Figure S16B), a probability that reaches a maximum close to GC=40%.

### 1.7. Phobius prediction assessment

Tools to predict signal peptide presence and transmembrane segments have been widely used for standard proteins but their efficiency in small proteins is still uncertain. To ensure our predictions were not biased, we performed two different tests to check whether the false positive predictions and sensitivity of Phobius is dependent on size.

For the first case, we ran Phobius on the 'decoy' dataset. From a total of 20,100 SEPs, 239 SEPs (1.18%) had a signal peptide and 942 (4.7%) had at least one transmembrane

segment. These numbers can be considered as the positives expected by chance. Thus, the observed values for the 36,311 predicted SEPs (9.7% and 15%, respectively) are higher than expected. This hypothesis was tested by Fisher's test after applying a central limit normalization, and returned significant p-values. This indicated that the observed values were higher than expected (p-value=1.5E-4 for signal peptide and 0.012 for transmembrane).

The second test aimed at evaluating the sensitivity of the signal peptide detection by Phobius in relation to the size of the protein studied.For this purpose, we subset annotated proteins of *M. pneumoniae* with size >200 aa and, starting from the 200 aa in the C'-terminus, sequentially removed amino acids 20 by 20 and computed the same prediction to check the percentage of proteins keeping the signal peptide/transmembrane segments. As expected, more than the 50% of the transmembrane segments are lost during this process as they are distributed along the protein sequence with no location specificity. This is different for the signal peptide, a sequence that is based on a motif within the first 16 to 30 N'- terminal amino acids. This motif must have 5 to 16 hydrophobic amino acids forming an alpha-helix and a fewer number of positively charged amino acids. With this in mind, we would expect to keep many of the signal peptide sequences until reaching a protein size of 30 amino acids. In fact, this is what we observe, as more than 80% of the proteins still have a signal peptide when only 40 aa are considered (Figure S11).

Both tests together indicate that in our predicted set there are more proteins with signal peptides than expected by chance and the sensitivity of Phobius for signal peptide detection is >80% for proteins with a size >30 aa. Taking this into account, we can trust

the predicted peptide signals for the predicted SEPs, expecting at most, that 1.2% could be positives for a signal peptide by chance.

## 2. SUPPLEMENTARY FIGURES LEGENDS

**Figure S1. Co-elution of labelled and endogenous peptides.** MS1 Area Extraction of labelled and endogenous peptides ELGIHDFDENLNEQSLLK, GGEATTSLTTNDPALK, FVEPADFLGIR, VDLAQTLPGR, DETNICSQSLK, TSQITQQTTTNEK, ALALVELIK, and ILIKSPPSGLK, Skyline software.

**Figure S2. MSMS spectra of labelled peptides.** MSMS spectra of labelled peptides ELGIHDFDENLNEQSLLK, GGEATTSLTTNDPALK, FVEPADFLGIR, VDLAQTLPGR, DETNICSQSLK, TSQITQQTTTNEK, ALALVELIK, and ILIKSPPSGLK, Proteome Discoverer software.

**Figure S3. Responsiveness of annotated proteins.** Evaluation of the responsiveness to shotgun MS of the UTPs derived from standard annotated proteins, annotated SEPs and decoy proteins using PeptideSieve. In blue, proteins detected by MS. In orange, proteins not detected by MS.

**Figure S4. Overlapping landscapes for negative-scored smORFs that had a signal in other methodologies in *M. pneumoniae*.** A) Pie chart representing the percentage of smORFs with negative score by RanSEPs but signal by transcriptomics. We considered different annotation types (colors) and consider a smORF to be overlapping when its

50% of length is part of the genomic annotations considered. As can be noticed, most of the smORFs′ transcription signals can be explained by their overlap with annotated genes. B) Same analysis than in the previous panel A but considering this time conserved smORFs with negative score by RanSEPs. In this case, annotated genes in the opposite strand and functional RNAs are the most populated groups.

**Figure S5. Ribo-Seq and MS integration for SEPs in *E. coli*.** Statistical analysis was supported by Mann-Whitney rank test to extract a p-value evaluating the difference in RCV between proteins grouped by their number of UTPs: Annotated with no UTPs detected, SEPs with 1 UTP and SEPs with ≥2 UTPs. We report the size of each subgroup on the top part of the figures (N) and the percentage of those groups that were previously annotated by NCBI. RCV is significantly higher for those SEPs appearing with 2 UTPs or more than those detected with 1 UTP or with no MS signal. Annotations with RCV=0.0 are filtered and percentage within the box represents the percentage of values in that class that are kept in the comparative.

**Figure S6. Classification statistics in *M. pneumoniae* using 5 iterations of RanSEPs. A) Score thresholds in prediction.** Histogram representing the distribution of scores provided by RanSEPs depending on the set of proteins considered. Dashed lines represent the 95$^{th}$ percentiles of each distribution used as a threshold in the prediction. **B) Precision recall curve.** Relationship between precision (positive predicted values = TPs / Total positives) and the recall (true positive rate) in the classification of proteins by RanSEPs. **C) ROC curve**. Relationship between the true positive rate (TPR) and the false positive rate (FPR). Average area under the curve (AUC) combining the 50 single

predictions.

**Figure S7. Precision-Recall curve for the method comparative.** Relationship between precision (positive predicted values = TPs / Total positives) and the recall (true positive rate) in the classification of proteins by different methodologies using true and false SEPs (N=1140).

**Figure S8. Computational performance comparison.** Comparison of RanSEPs, glimmer and prodigal in terms of **A)** time required per prediction in RanSEPs with 1, 5 and 25 iterations, glimmer and prodigal (both using default settings) and **B)** CPU usage where RanSEPs is divided into two steps: Blast searches and prediction per iteration. X axis represents 8 different genome sizes with the name of the species associated and Y axis represent time in seconds and percentage of CPU in use.

**Figure S9. Categories of former ncRNAs re-annotated as proteins.** Barplot including the percentage of former ncRNAs that could actually be proteins. 'PO' stands for Partial Overlap; 'SS' for sense ncRNAs; 'AS' for antisense ncRNA; and 'IG' for intergenic. Data integrated from 11 bacterial species (N explored=8056, N represented=273).

**Figure S10. Conservation landscape for all the novel SEPs scored as positive by RanSEPs.** Barplot relating the number of SEPs and the number of organisms in which they can be found. 107 SEPs were not conserved in any organism while the rest can be found, on average, in 15 other organisms. 109 total bacterial species considered.

**Figure S11. Sensitivity of Phobius to detect signal peptide and transmembrane segments.** We selected annotated proteins with a size >200 amino acids from the

database generated with 109 bacterial species. We sequentially removed 20 amino acids from the N'-terminus of this proteins, and checked what percentage of them (x axis) kept the signal peptide/transmembrane segments. As expected, transmembrane segments (blue) are reduced at a higher rate than signal peptides as they are distributed along the sequence. However, signal peptide detection only depends on the last 16-30 amino acids of the N'-terminus so more than the 80% kept their signal peptides until the threshold of ~40 is surpassed.

**Figure S12. Exploration of detection by different techniques of 17,818 smORFs in *M. pneumoniae.* A)** A Venn diagram showing the landscape of putative SEPs detected by RNAseq, shotgun MS, conservation (dark to lighter blue), and/or RanSEPs (orange). The pies (grey color) indicate the percentage of SEPs overlapping with other transcriptionally active regions of the genome (bottom), or the percentage of SEPs conserved in other genomes (top). **B)** A comparison between the number of high-responsive UTPs (HR_UTPs) assigned by ESPPredict to RanSEPs-positive proteins (standard and SEPs) for shotgun MS-detected and -undetected proteins (p-value=0.002). **C)** Evaluation of the responsiveness to shotgun MS of the UTPs derived from proteins positively scored by RanSEPs and from decoy proteins using ESPPredict.

**Figure S13. Exploration of detection by different techniques of 1,292 ORFs in *M. pneumoniae.*** A) Venn diagram of the detection of annotated standard proteins by shotgun MS. B) Venn diagram of the detection of novel standard proteins by shotgun MS.

**Figure S14. Comparison of different genome annotation tools for *M. pneumoniae*.** A) The histogram represents the percentage of annotated proteins that are correctly detected by six different software. B) A map comparing the detection of validated novel proteins by different annotation tools and conservation (columns 1 to 7), with the results from shotgun MS experiments (column 8). Each colored bar represents a new protein predicted by the corresponding method. The color code in the MS column is: validated by non-targeted MS (gray), also validated with C13 labeled peptides (light orange), only validated by C13 labeled peptides (dark orange), and detected with one UTP in non-targeted MS but not found using C13 labeled peptides (black).

**Figure S15. Score comparative between DISCO-Bac and RanSEPs.** Comparison of scores returned by the DISCO-Bac software and RanSEPs for a set of 17 SEPs tested by targeted MS in *Helicobacter pylori*. Each dot is one of the considered SEPs Orange are SEPs validated by labelled peptides and blue are SEPS not validated by labelled peptides. Dashed red lines represent the thresholds established by each methodology to assign the coding category to a smORF.

**Figure S16. Probability of finding a SEP based on GC content A)** Probability of finding START or STOP codons based on the % of GC. **B)** Probability of finding a SEP protein based on the % of GC. CTT stands for Codon Translation Table.

# Figure S1

## Co-elution of labelled and endogenous peptides.

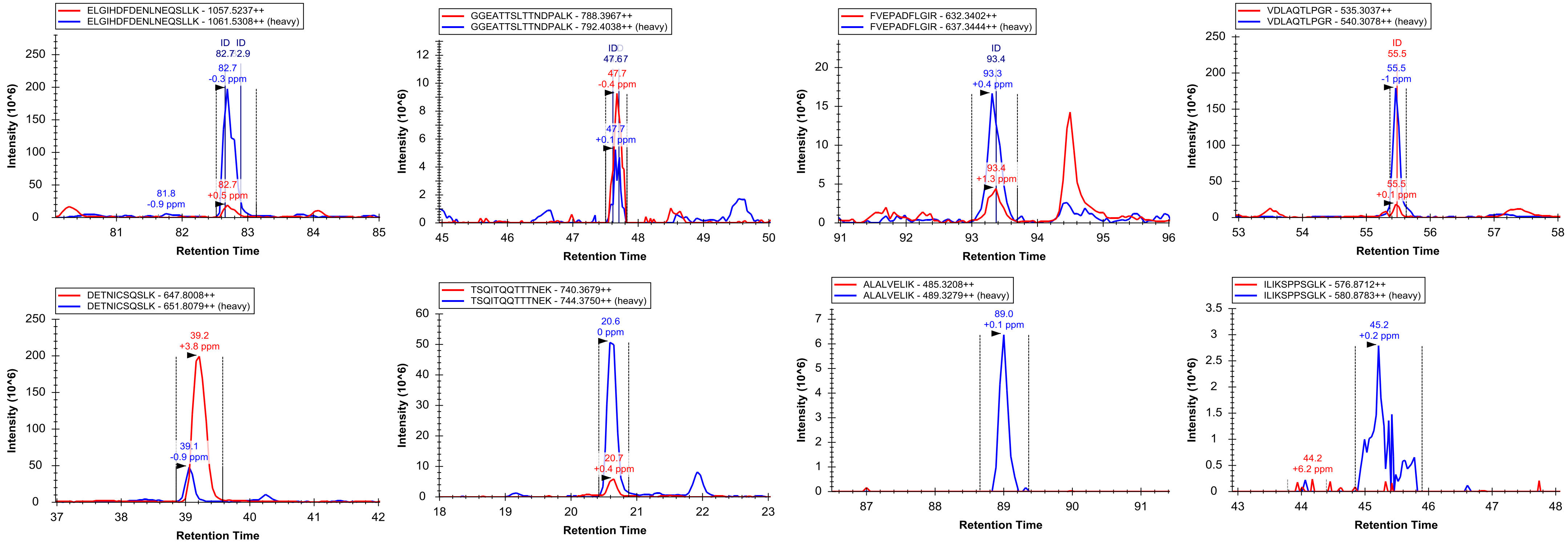

# Figure S2

## MSMS spectra of labelled peptides.

### ELGIHDFDENLNEQSLLK

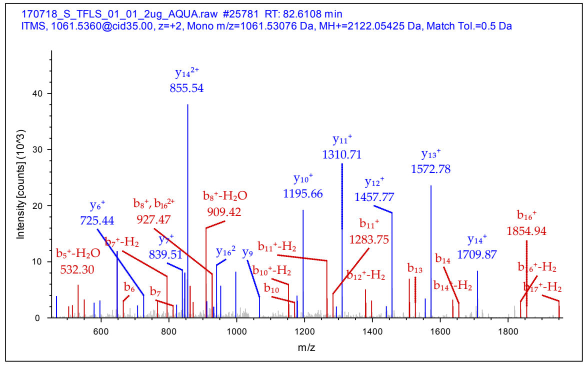


### GGEATTSLTTNDPALK

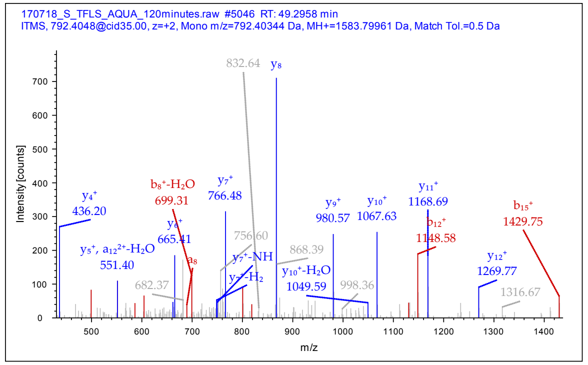


### FVEPADFLGIR

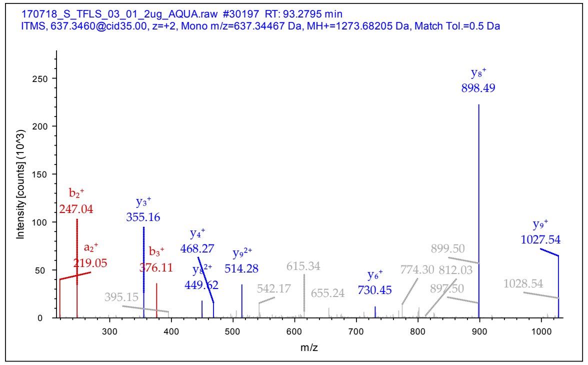


### VDLAQTLPGR

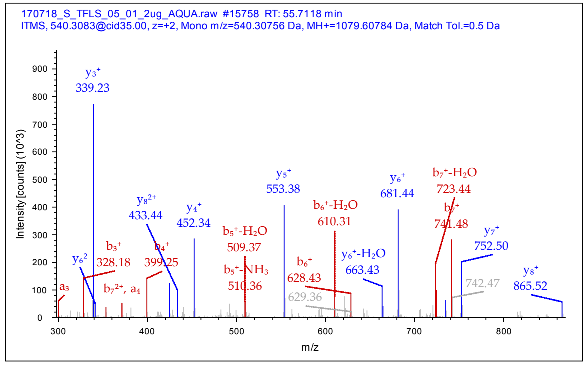


### DETNICSQSLK

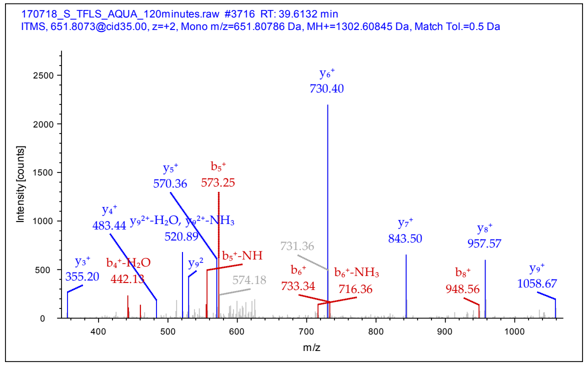


### TSQITQQTTTNEK

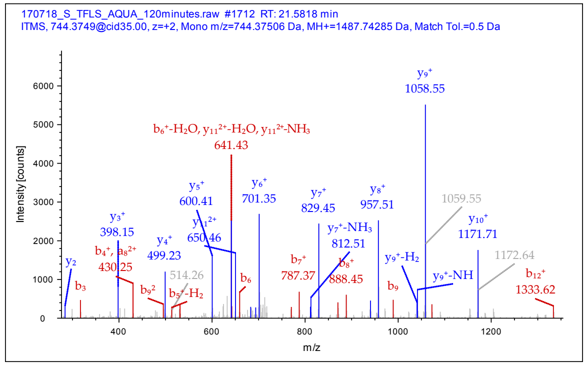


### ALALVELIK

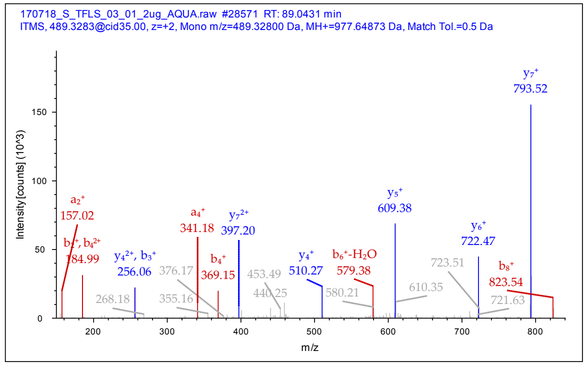


### ILIKSPPSGLK

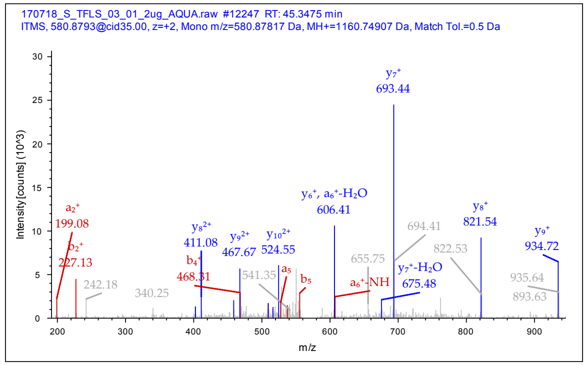

# Figure S3

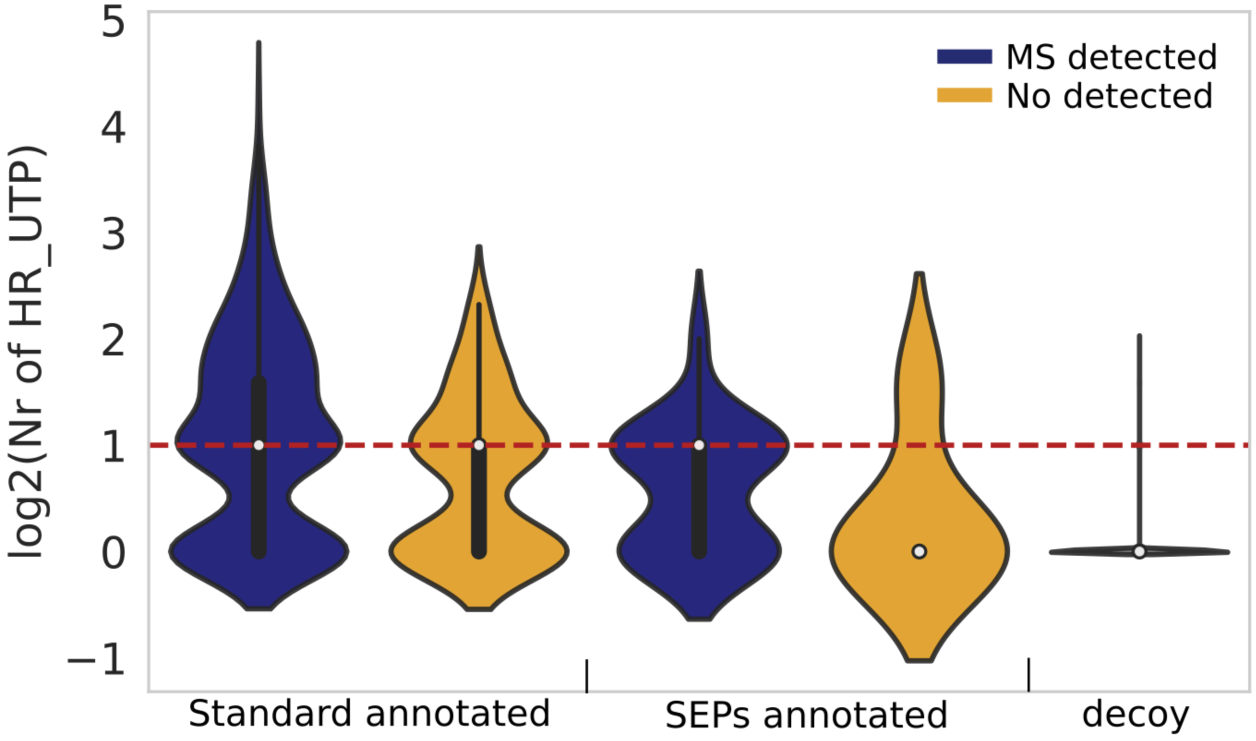

MS detected
No detected
log2(Nr of HR_UTP)
5
4
3
2
1
0
−1
Standard annotated
SEPs annotated
decoy

# Figure S4

A

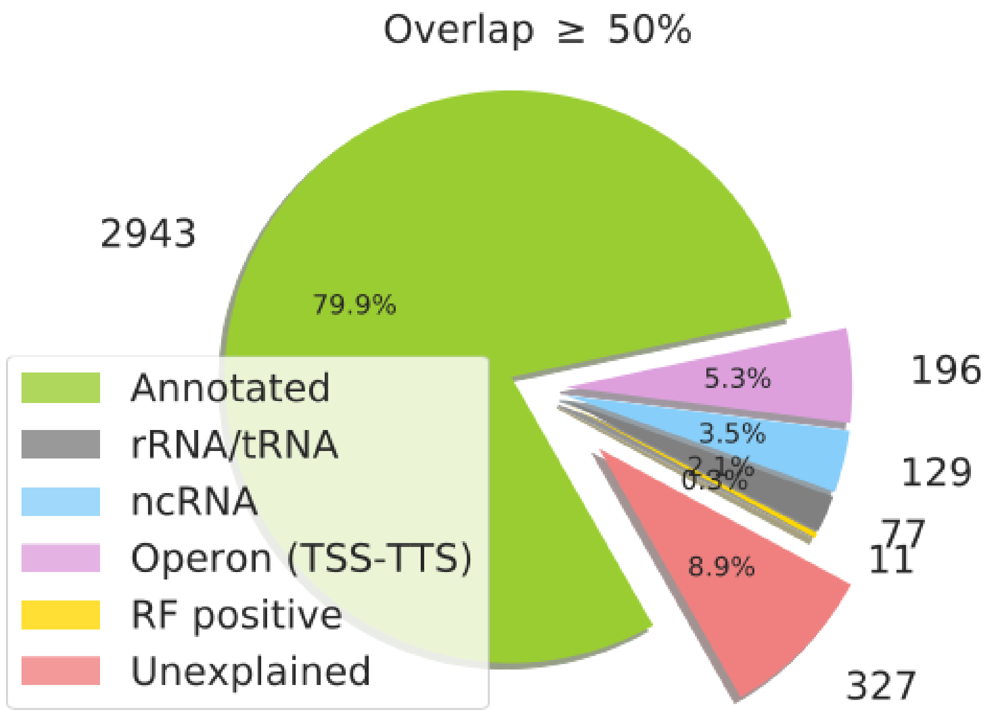
Overlap ≥ 50%
2943
79.9%
196
5.3%
129
3.5%
2.1%
0.3%
77
11
8.9%
327
Annotated
rRNA/tRNA
ncRNA
Operon (TSS-TTS)
RF positive
Unexplained

B

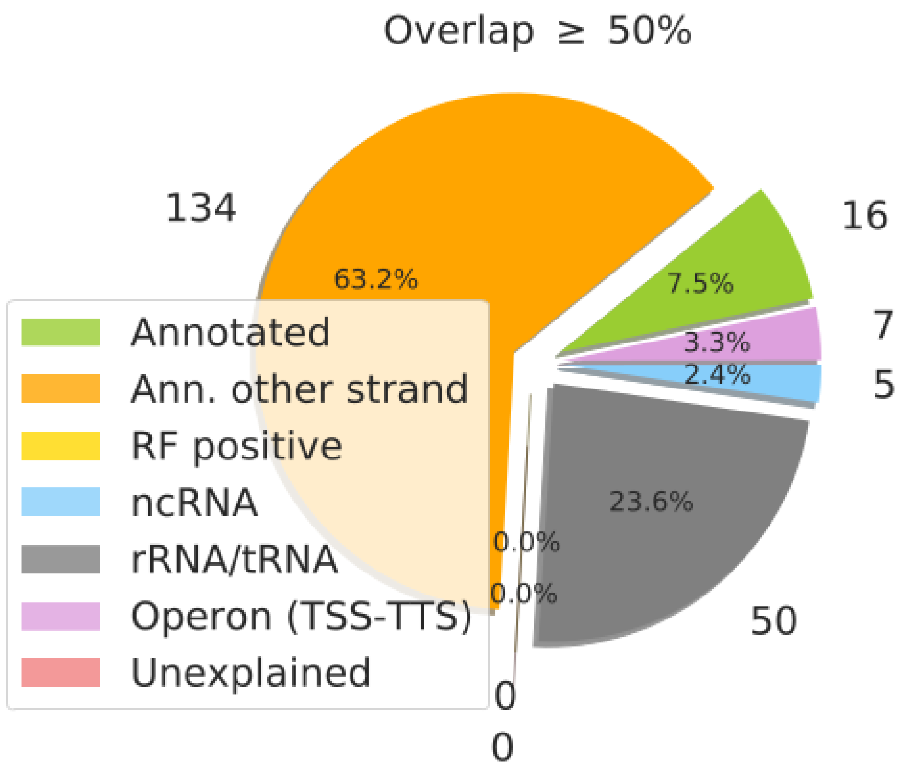
Overlap ≥ 50%
134
63.2%
16
7.5%
7
3.3%
5
2.4%
23.6%
0.0%
0.0%
50
0
0
Annotated
Ann. other strand
RF positive
ncRNA
rRNA/tRNA
Operon (TSS-TTS)
Unexplained

# Figure S5

A

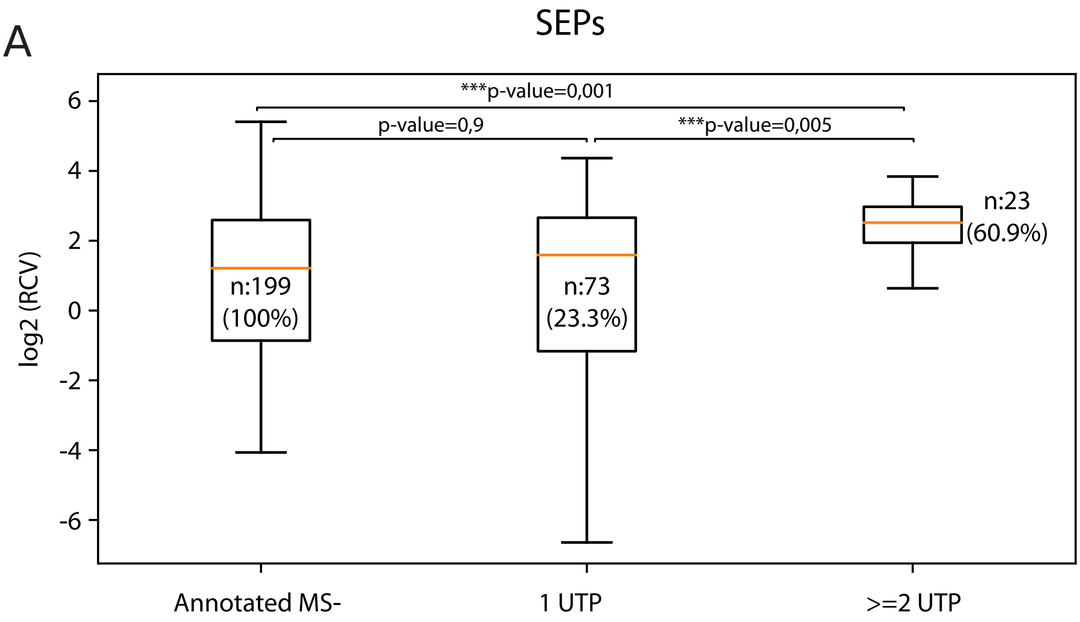
SEPs
***p-value=0,001
p-value=0,9
***p-value=0,005
log2 (RCV)
6
4
2
0
-2
-4
-6
n:199
(100%)
n:73
(23.3%)
n:23
(60.9%)
Annotated MS-
1 UTP
>=2 UTP

# Figure S6

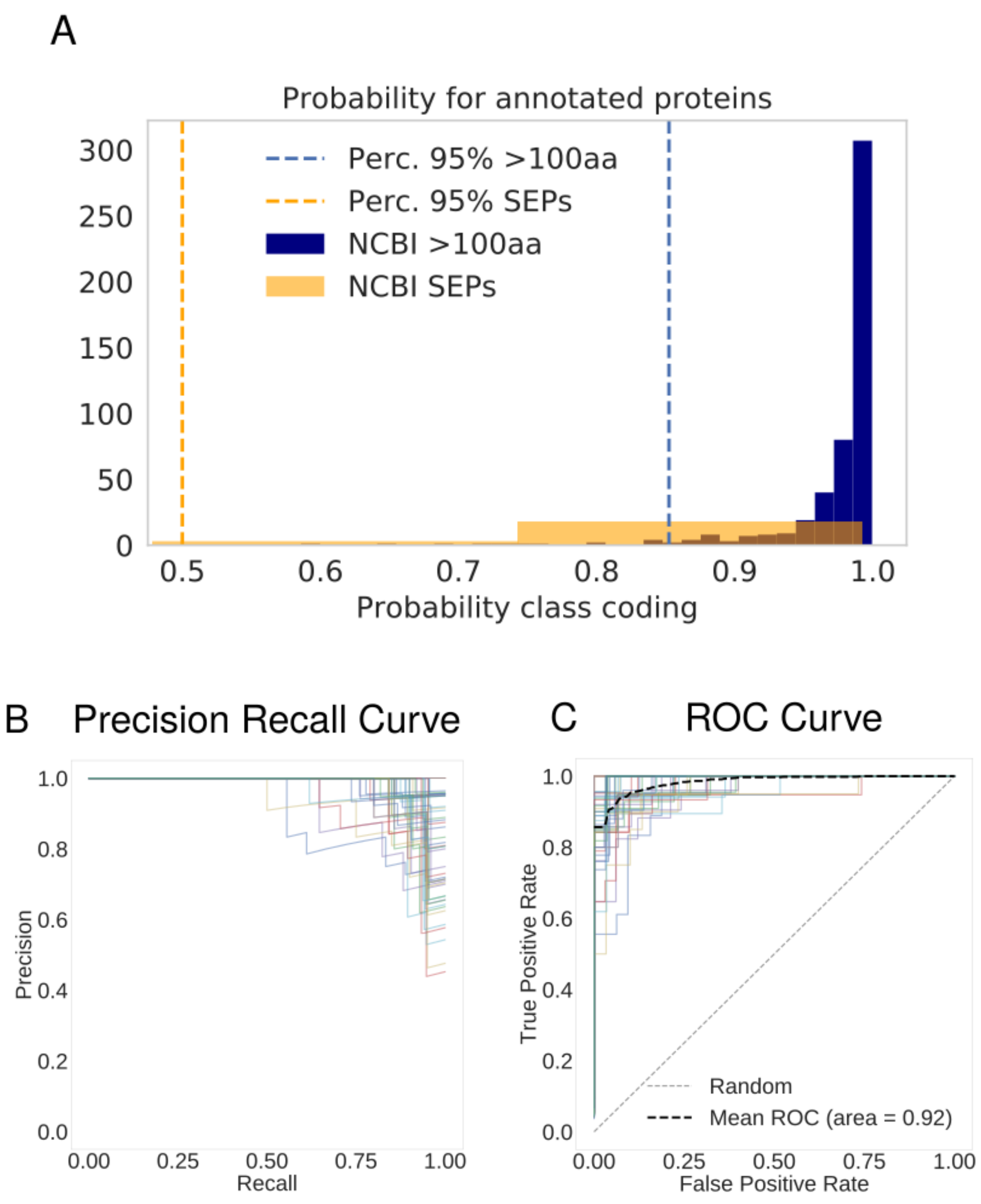
A
Probability for annotated proteins
Perc. 95% >100aa
Perc. 95% SEPs
NCBI >100aa
NCBI SEPs
300
250
200
150
100
50
0
0.5
0.6
0.7
0.8
0.9
1.0
Probability class coding
B
Precision Recall Curve
Precision
Recall
1.0
0.8
0.6
0.4
0.2
0.0
0.00
0.25
0.50
0.75
1.00
C
ROC Curve
True Positive Rate
False Positive Rate
Random
Mean ROC (area = 0.92)

# Figure S7

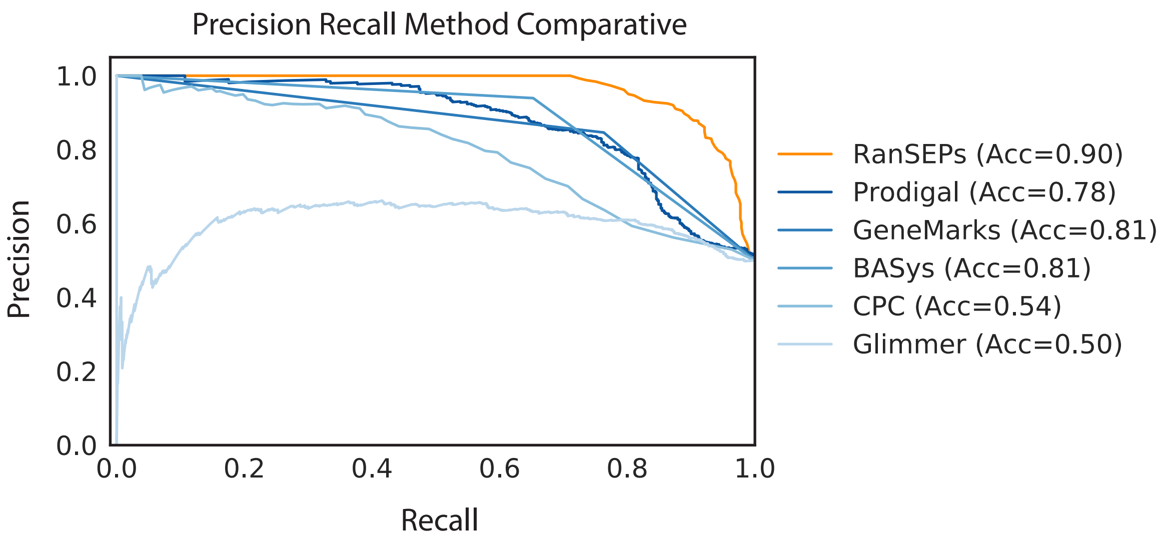

Precision Recall Method Comparative
Precision
Recall
1.0
0.8
0.6
0.4
0.2
0.0
0.0
0.2
0.4
0.6
0.8
1.0
RanSEPs (Acc=0.90)
Prodigal (Acc=0.78)
GeneMarks (Acc=0.81)
BASys (Acc=0.81)
CPC (Acc=0.54)
Glimmer (Acc=0.50)

# Figure S8

A

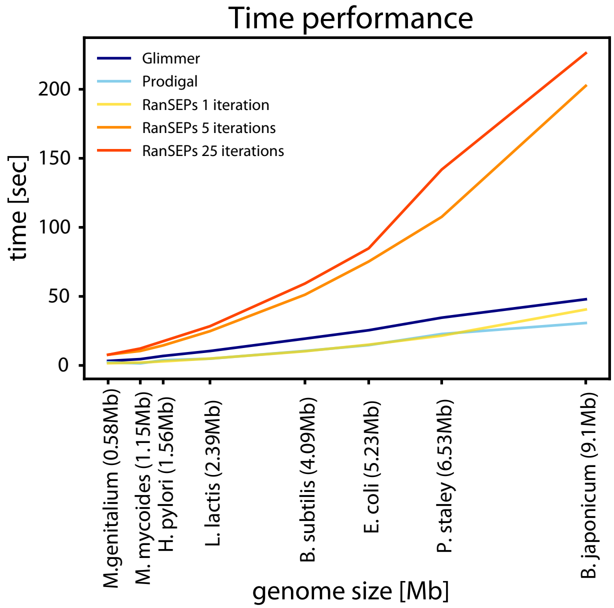
Time performance
Glimmer
Prodigal
RanSEPs 1 iteration
RanSEPs 5 iterations
RanSEPs 25 iterations
time [sec]
0
50
100
150
200
M.genitalium (0.58Mb)
M. mycoides (1.15Mb)
H. pylori (1.56Mb)
L. lactis (2.39Mb)
B. subtilis (4.09Mb)
E. coli (5.23Mb)
P. staley (6.53Mb)
B. japonicum (9.1Mb)
genome size [Mb]

B

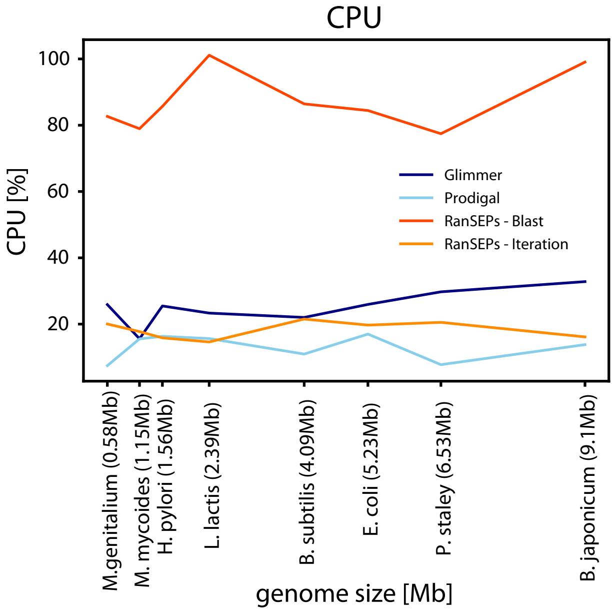
CPU
Glimmer
Prodigal
RanSEPs - Blast
RanSEPs - Iteration
CPU [%]
20
40
60
80
100
M.genitalium (0.58Mb)
M. mycoides (1.15Mb)
H. pylori (1.56Mb)
L. lactis (2.39Mb)
B. subtilis (4.09Mb)
E. coli (5.23Mb)
P. staley (6.53Mb)
B. japonicum (9.1Mb)
genome size [Mb]

# Figure S9

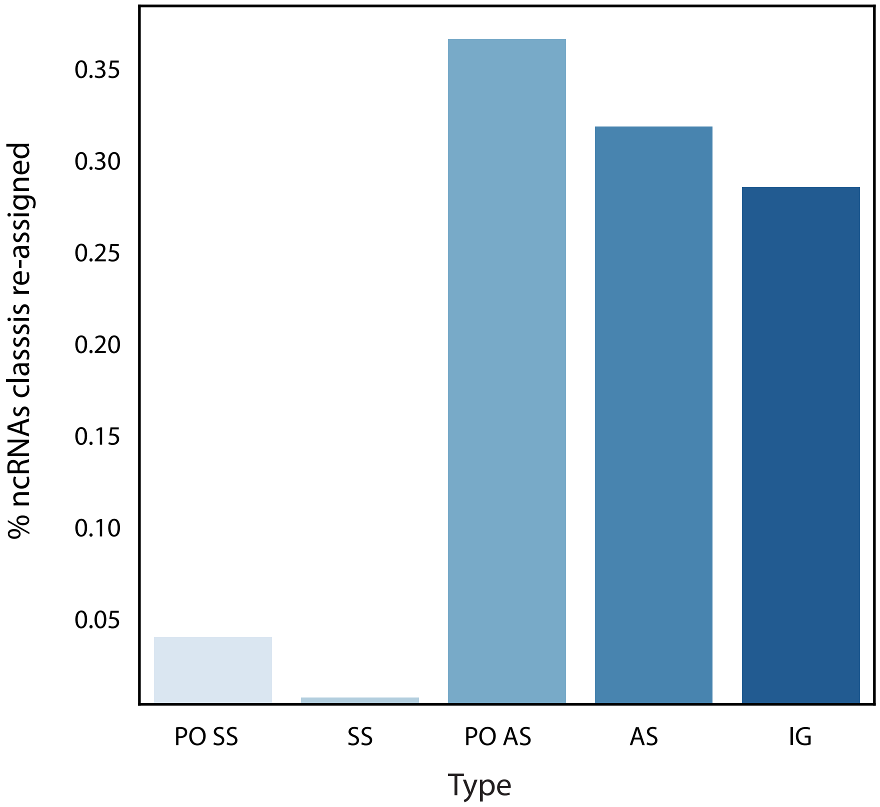
0.35
0.30
0.25
0.20
0.15
0.10
0.05
% ncRNAs classis re-assigned
PO SS
SS
PO AS
AS
IG
Type

Figure S10

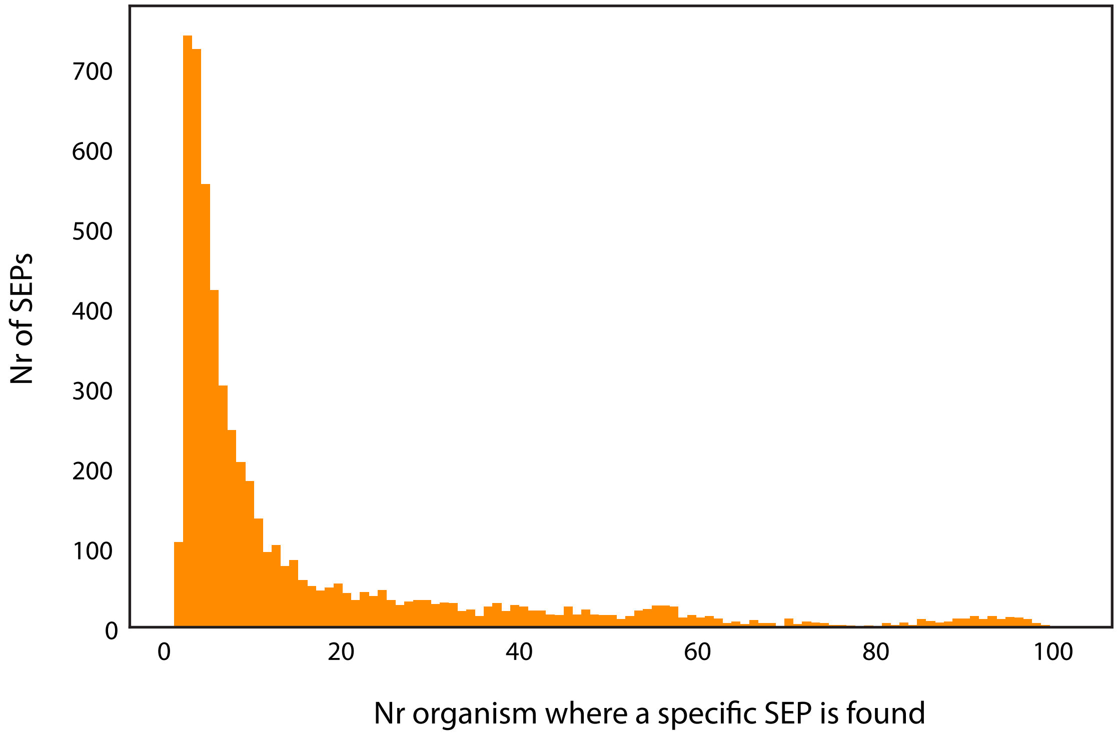

Nr of SEPs
700
600
500
400
300
200
100
0
0
20
40
60
80
100
Nr organism where a specific SEP is found

# Figure S11

A

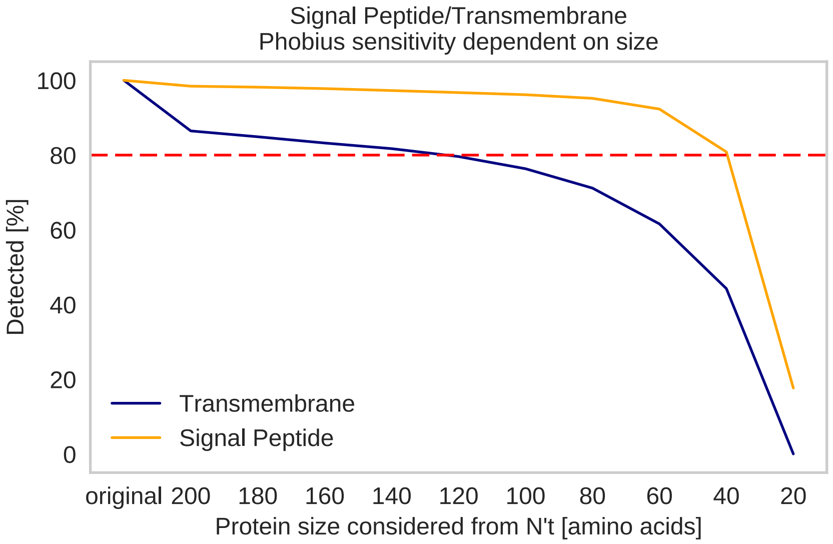

Signal Peptide/Transmembrane
Phobius sensitivity dependent on size
Detected [%]
100
80
60
40
20
0
Transmembrane
Signal Peptide
original
200
180
160
140
120
100
80
60
40
20
Protein size considered from N't [amino acids]

Figure S12

A

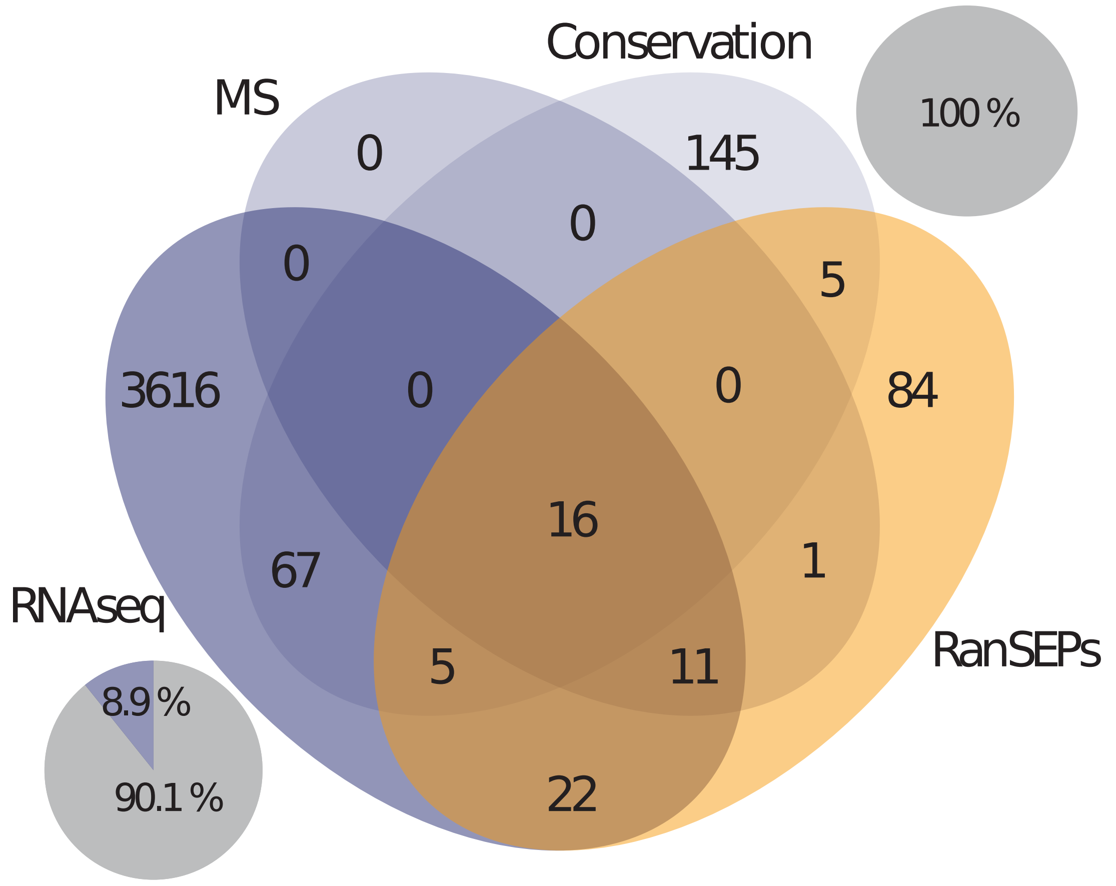


B

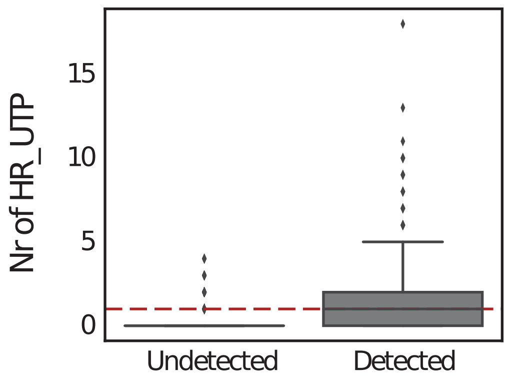


C

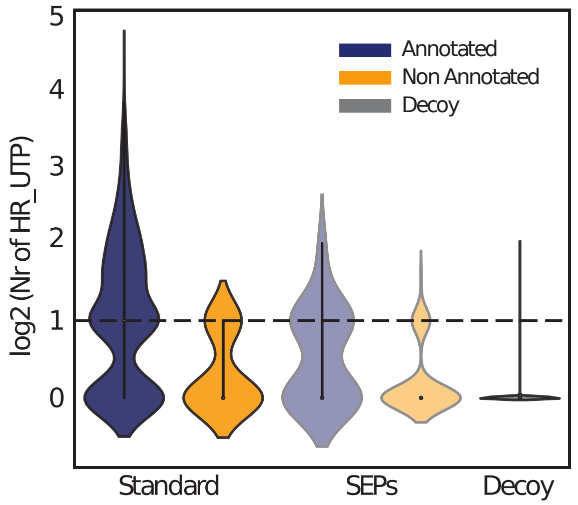

Figure S13

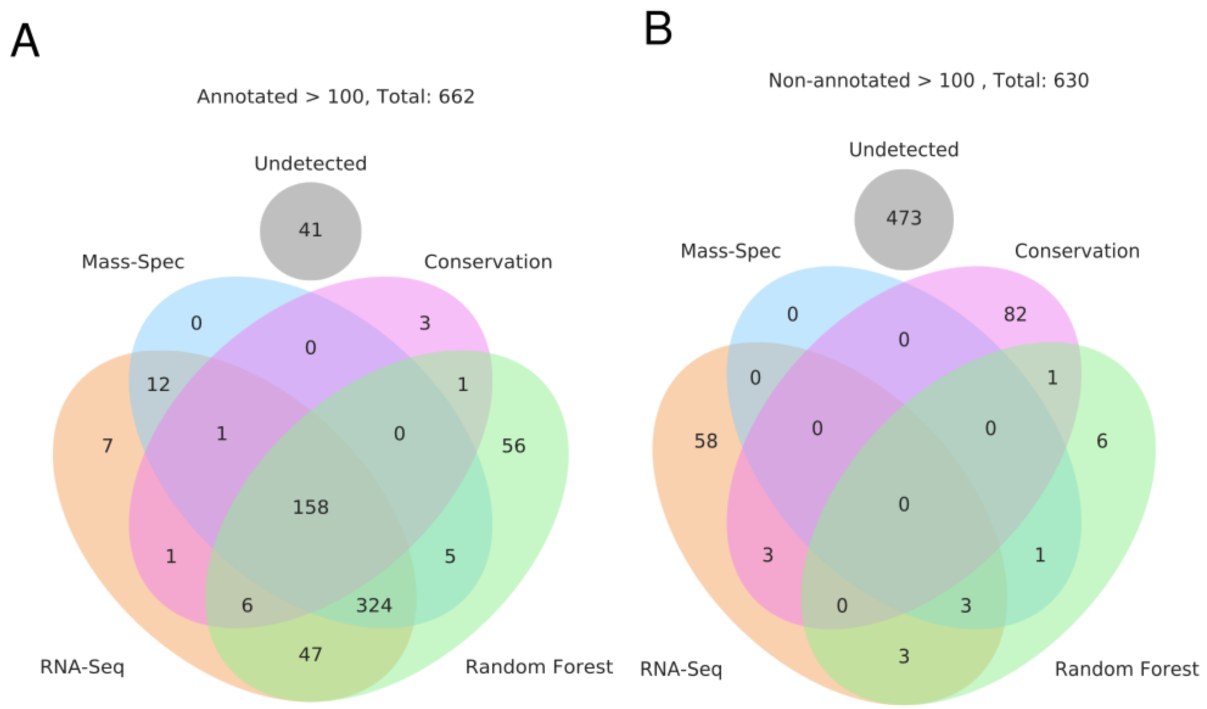
A
Annotated > 100, Total: 662
Undetected
41
Mass-Spec
Conservation
0
0
3
12
1
7
1
0
56
158
1
5
6
324
47
RNA-Seq
Random Forest
B
Non-annotated > 100 , Total: 630
Undetected
473
Mass-Spec
Conservation
0
82
0
0
1
58
0
0
6
0
3
1
0
3
3
RNA-Seq
Random Forest

# Figure S14

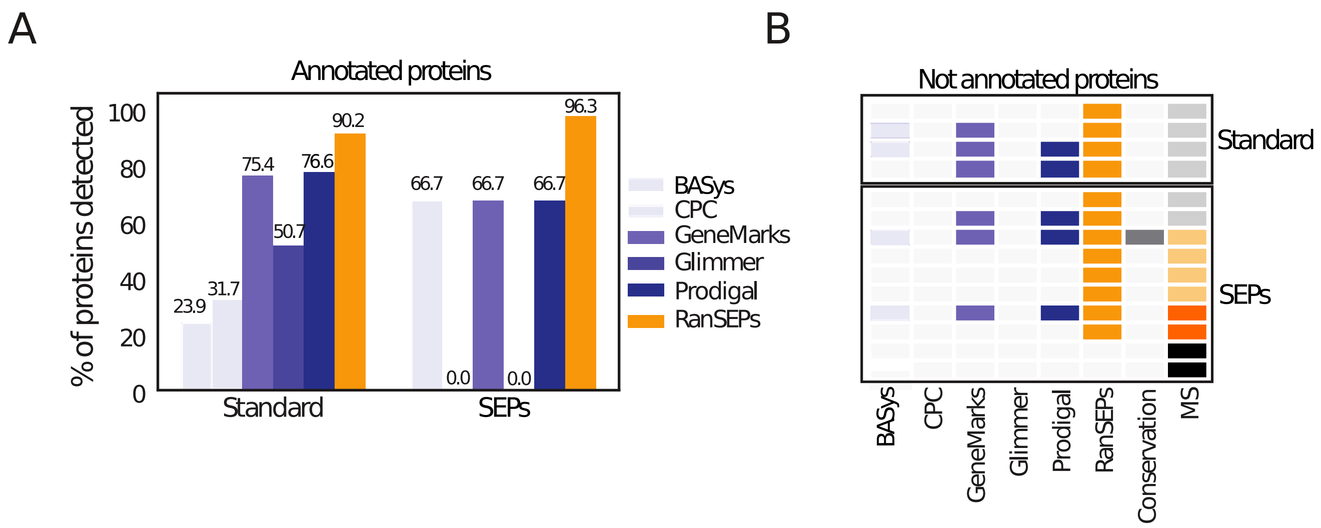
A
Annotated proteins
% of proteins detected
100
80
60
40
20
0
23.9
31.7
75.4
50.7
76.6
90.2
66.7
0.0
66.7
0.0
66.7
96.3
Standard
SEPs
BASys
CPC
GeneMarks
Glimmer
Prodigal
RanSEPs
B
Not annotated proteins
Standard
SEPs
BASys
CPC
GeneMarks
Glimmer
Prodigal
RanSEPs
Conservation
MS

Figure S15

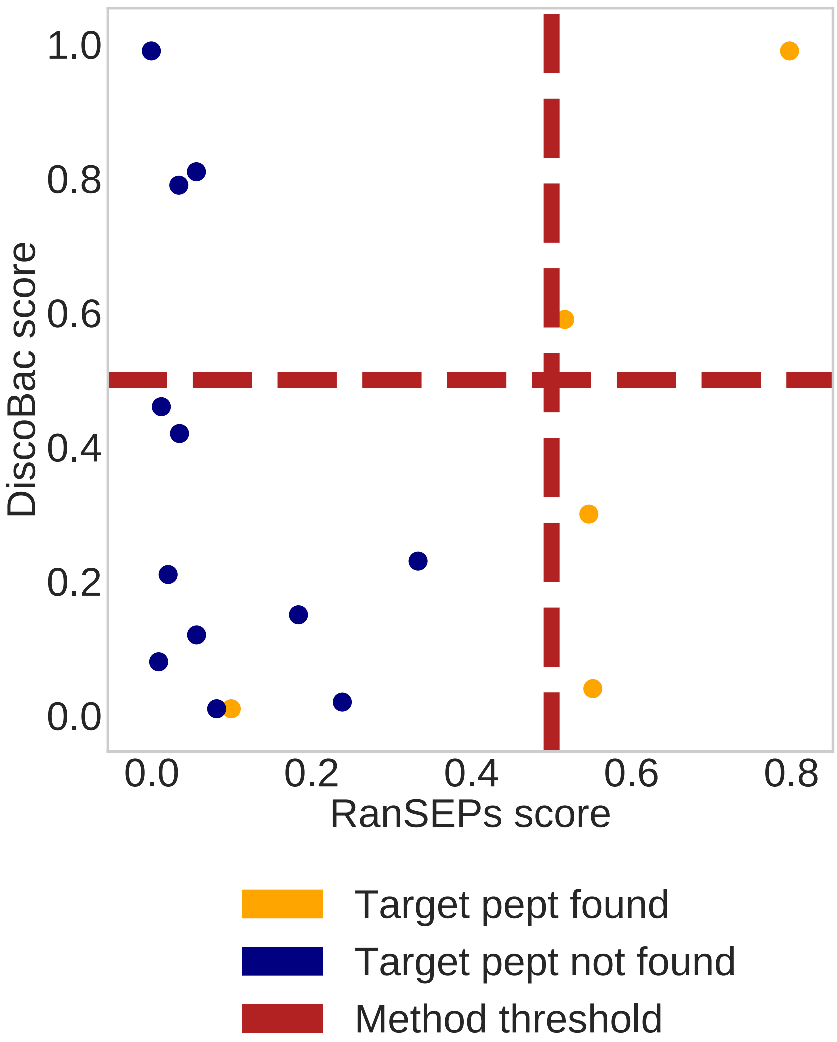

1.0
0.8
0.6
0.4
0.2
0.0
DiscoBac score
0.0
0.2
0.4
0.6
0.8
RanSEPs score
Target pept found
Target pept not found
Method threshold

Figure S16

A

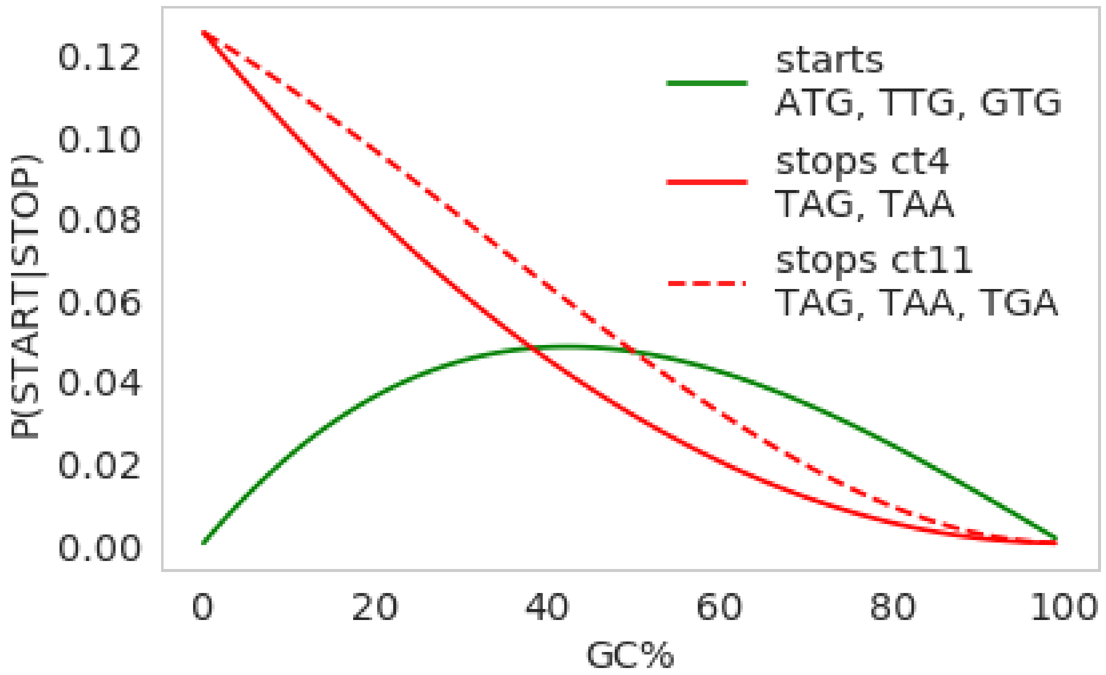


B

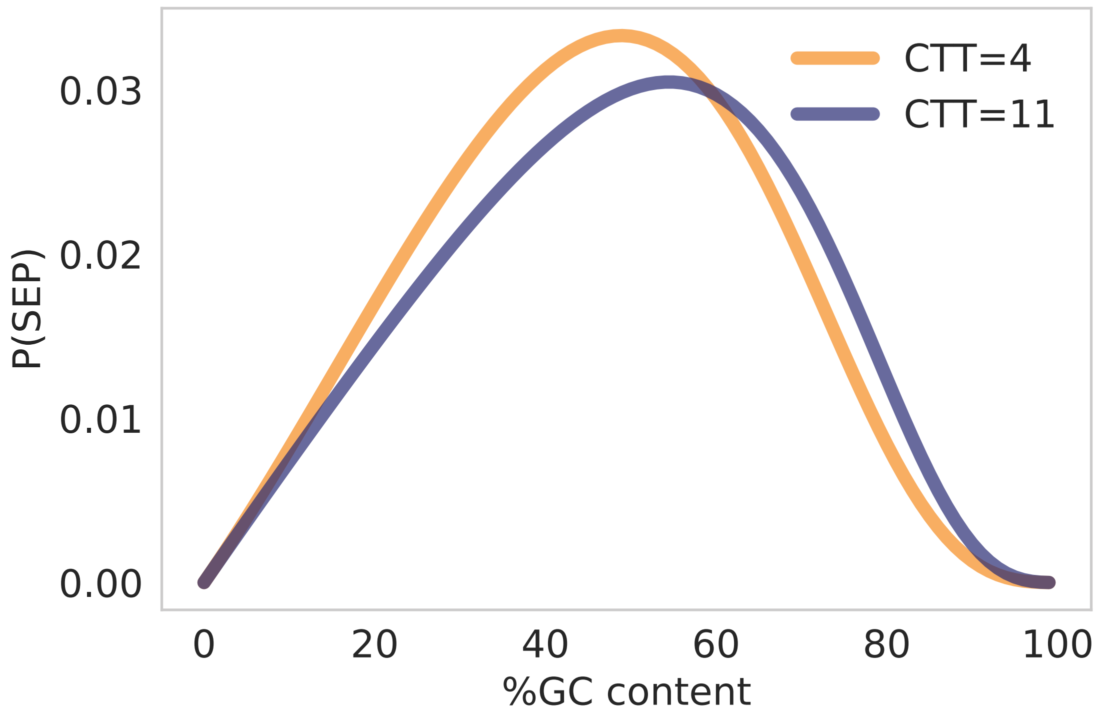

Supplement: Supplementary file 1 — Appendix [file MSB-15-e8290-s001.pdf]
